# What happens next?: a claims database study of second-line pharmacotherapy in patients with major depressive disorder (MDD) who initiate selective serotonin reuptake inhibitor (SSRI) treatment

**DOI:** 10.1186/1744-859X-13-8

**Published:** 2014-03-19

**Authors:** Susan Ball, Peter Classi, Ellen B Dennehy

**Affiliations:** 1 Eli Lilly and Company, Indianapolis, IN 46285, USA; 2 Indiana University School of Medicine, Indianapolis, IN 46202, USA; 3 Purdue University, West Lafayette, IN 47907, USA

**Keywords:** Treatment, SSRI, Prescription, Major depressive disorder

## Abstract

**Background:**

The objective of this research was to examine treatment patterns and health-care costs associated with second-step pharmacotherapy in patients with major depressive disorder (MDD) who initiated monotherapy with a selective serotonin reuptake inhibitor (SSRI) in 2010.

**Methods:**

This claims database study analyzed patients diagnosed with MDD who were prescribed a monotherapy SSRI, with the first prescription identified as the index date. Patients were required to be ≥18 years old, to have continuous insurance coverage from 1 year prior (pre-index) through 1 year post (post-index) from the index date, and to have not received an antidepressant in the pre-index period. The analyses are descriptive of the patient characteristics, initial SSRI prescribed, most commonly prescribed second-step therapies, and annualized health-care costs.

**Results:**

The identified patients (*N* = 5,012) were predominantly female (65.2%) with a mean age of 41.9 years. The most frequent index SSRIs were citalopram (30.1%) and sertraline (27.5%), and 52.9% of patients were prescribed a second-step pharmacotherapy during the post-index period. Add-on therapy occurred twice more frequently than switching treatments, with either anxiolytics (40.2%) or antidepressants (37.1%) as the most common classes of add-on pharmacological therapies. Patients who added a second medication or switched therapies had higher annualized medical costs compared with patients who continued their index SSRI or discontinued treatment.

**Conclusions:**

For patients who were initially treated with an SSRI therapy, approximately half were prescribed a second-step treatment. In this comprehensive claims analysis, many of these patients experienced the addition of second medication, rather than switching to a new therapy. Given the type of medications used, it is possible that second-step interventions were targeted toward resolution of residual symptoms; however, this work is limited by the use of claims data without information on dosing or clinical symptoms, side effects, or response. Findings from this study set the expectation that physicians and patients will most likely need to partner for additional interventions in order to achieve remission.

## Background

For adults in the USA, the 12-month prevalence rate of major depressive disorder (MDD) has been estimated at 8.3% with a lifetime prevalence rate of 19.2% [1]. Similar rates across other countries indicate that MDD is a global disease. Indeed, in the World Health Organization (WHO) Global Burden of Disease 2010 study, mental and substance use disorders were the fifth leading disorders of global disability-adjusted life years, with MDD accounting for 40.5% of this disability [2]. The burden of MDD is also expressed through substantial direct and indirect costs. In 2000, the total economic burden of treating depression in the USA was $83.1 billion. However, only 31% of these costs include direct treatment ($26.1 billion); the majority of the indirect costs involved workplace losses, with the remainder involving costs attributed to suicide [3].

In this context, treatment of MDD becomes imperative. Treatment guidelines recommend both pharmacotherapy and psychotherapy for MDD [4]. From a pharmacological perspective, selective serotonin reuptake inhibitors (SSRIs) are recommended as the first-line pharmacological intervention due to their favorable adverse event profile compared with other antidepressants and generic availability [4]. Ideally, with treatment, a patient would achieve remission of MDD, defined as being free or nearly free of symptoms. However, achievement of remission may be difficult to obtain with monotherapy treatment. For example, in the National Institute of Mental Health (NIMH)-funded Sequenced Treatment Alternatives to Relieve Depression (STAR*D) study, approximately one third of patients achieved remission during initial treatment with citalopram monotherapy, resulting in nearly two thirds of patients needing additional treatment interventions [5].

When faced with inadequate or partial response to an initial trial with an SSRI, physicians and patients often discuss next whether to add another medication or switch to a different therapy [6]. Although there is support for the efficacy of specific agents, the evidence to guide choice of second-step treatment interventions for individual patients, in general, is not clearly defined in current treatment guidelines [7-9]. For patients who have demonstrated a partial, positive response from an initial therapy, it may be more beneficial to add to that therapy, whereas for initial therapy associated with non-response or significant adverse effects, switching is often a recommended second step [7-9]. An important component of this decision making should be consideration of the patient’s preference about his or her treatment [10]. In the STAR*D study, patients could select the strategy, although not the specific agent, in the second step of treatment. Patients had clear preference about the acceptability of add-on therapy or switching, and few were willing to accept both types of interventions. Preference for the type of second-step intervention was related to the initial efficacy response to the monotherapy as well as the burden of adverse events associated with the treatment [11].

Examining treatment patterns after initial monotherapy with a SSRI can provide insight into the unmet medical needs of patients with MDD as well as the current state of second-step treatment practices. Accordingly, the objective of this research was to examine treatment patterns and outcomes of second-line pharmacotherapy among patients with MDD who initiated monotherapy with an SSRI. Patients were tracked for 1 year after they initiated SSRI therapy, and information was obtained on demographics, second-line pharmacological treatments, and direct health-related costs.

## Methods

Data for this study came from the Thomson Reuters MarketScan® Commercial Claims and Encounters (CCAE) Database from the USA. This claims database is fully compliant with the United States Health Insurance and Portability Act (HIPAA) privacy requirements while capturing person-specific clinical utilization, expenditures, and enrollment across inpatient, outpatient, prescription drug, and behavioral services. The database includes large employers, health plans, government organizations, and public organizations, covering approximately 100 payers and more than 500 million claims records. Paid claims and encounter data are linked to detailed patient information across sites and types of providers over time. Data records examined for this study spanned the years 2009 through 2011.

For inclusion in this study, a patient was required to be diagnosed with MDD in 2010–2011. Furthermore, to ensure that patients were reliably diagnosed with MDD, they needed to have at least one additional diagnosis claim of MDD within 6 months following the initial diagnosis. Patients were required to have a prescription for an SSRI in 2010 (with the first prescription date identified as the index date), to be at least 18 years old at the index date, and to have continuous insurance coverage from 1 year before the index date (the pre-index period) through 1 year after the index date (the post-index period). Patients were excluded from the analysis if they received a prescription for any antidepressant in the pre-index period or if they initiated therapy on more than one antidepressant.

For patients who met these study criteria, the treatment pattern following the initial SSRI monotherapy was then categorized as ‘continuation’ , ‘switch’ , ‘add-on’ , and ‘discontinuation’. Continuation was defined as the use of SSRI therapy throughout the entire 1-year post-index period. Switching was defined as the discontinuation of SSRI treatment followed by the initiation of therapy with another psychotropic agent. Add-on was defined as adding another psychotropic agent during the prescription period of the initial SSRI. Discontinuation was defined as patients who stopped SSRI therapy prior to the end of the post-period without switching to any other psychotropic medications.

Patient demographics, including age, sex, region of residence, and type of insurance coverage, were analyzed for the overall cohort. General health was approximated using the Charlson comorbidity index [12], which measures 17 different categories of comorbidities and creates a score that is a proxy of 10-year mortality rates. Additionally, a chronic disease score was calculated as a measure of aggregate comorbidity based on current concomitant medication use [13]. Descriptive statistics were calculated for the type of initial SSRI therapy and the second-step intervention. Direct health-care costs associated with inpatient, outpatient, and medications were also calculated for each pattern of treatment intervention. For mean values, differences between the groups were examined using *t* statistics. When values were non-normally distributed (for example, costs), Wilcoxon tests were used to assess group differences. All analyses were conducted using SAS, version 9.2, and findings of *P* values ≤0.05 were considered statistically significant.

## Results

**Figure 1:**
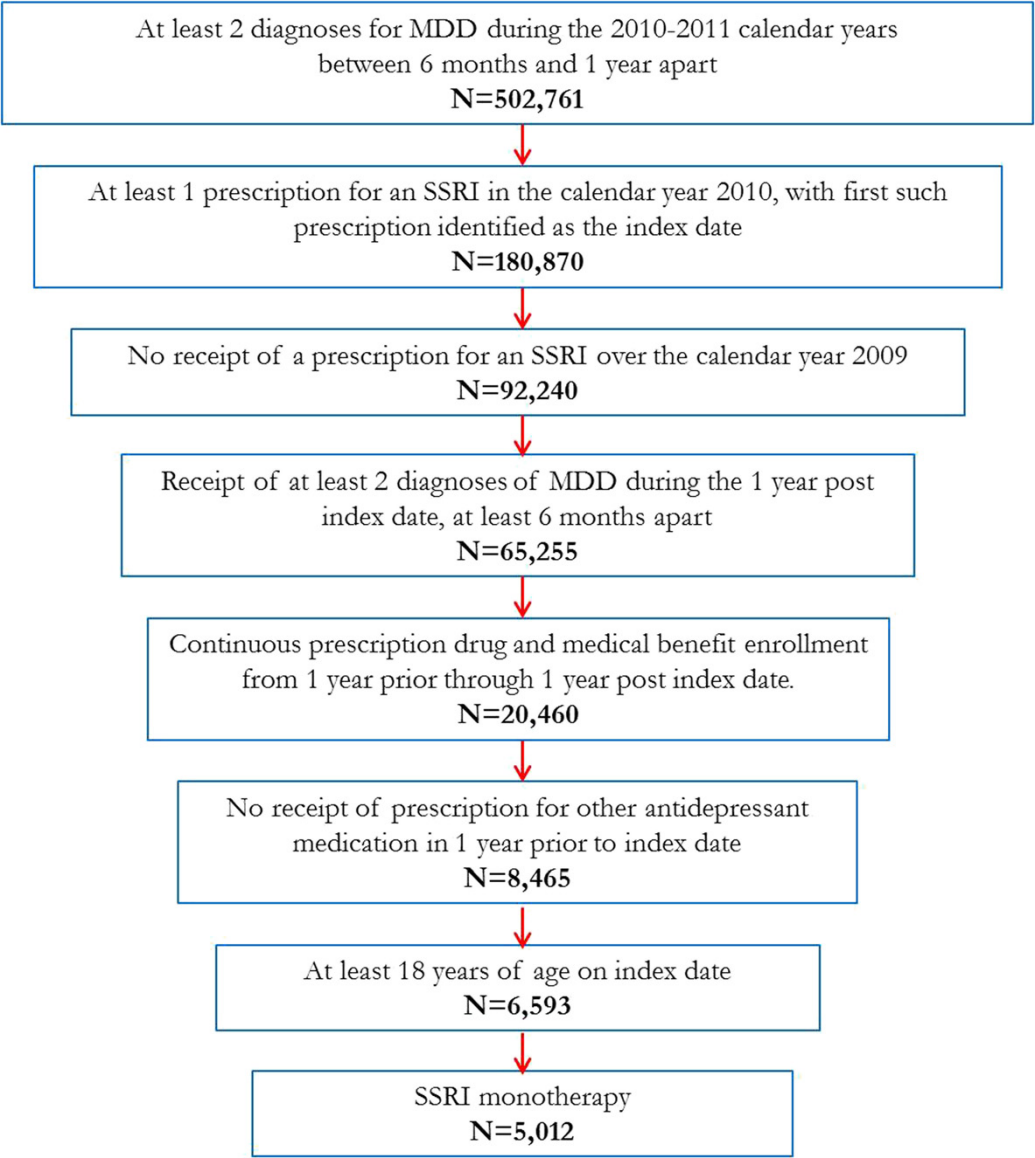
Funnel plot for inclusion and exclusion criteria and sample size within the database.

In the database, 5,012 patients met the inclusion criteria. For an attrition funnel diagram, refer to Figure 1. The mean age of patients was 41.9 years (standard deviation (SD) = 15.3 years), and approximately two thirds were female (65.2%). Patients were most commonly insured with a preferred provider organization (54.3%) and resided in the west (30.4%) or south (28.6%) regions of the USA. The mean Charlson comorbidity index score was 0.5, and the mean chronic disease score was 2.1 (see Table 1 for patient characteristics). The most frequently prescribed SSRI as a monotherapy was citalopram (30.1%), followed by sertraline (27.5%), escitalopram (18.7%), fluoxetine (17.9%), paroxetine (5.4%), and fluvoxamine (0.3%).

**Table 1:** Demographics of patients with MDD who receive initial treatment with SSRI monotherapy

**Characteristic**	**Initial treatment with SSRI (*N*** **= 5,012)**
Gender; *n* (%)	
Female	3,267 (65.2)
Male	1,745 (34.8)
Age; mean (SD)	41.9 (15.3)
Age distribution in years; *n* (%)	
18–24	773 (15.4)
25–34	948 (18.9)
35–44	1,164 (23.2)
45–54	1,110 (22.1)
55–64	722 (14.4)
65+	295 (5.9)
Plan type; *n* (%)	
CDHP	174 (3.5)
COMP	224 (4.5)
EPO	78 (1.6)
HMO	1,233 (24.8)
Non-capitated POS	449 (9.0)
POS with capitation	9 (0.2)
PPO	2,696 (54.3)
Missing/unknown	99 (2.0)
Region; *n* (%)	
Northeast	718 (14.3)
North central	1,324 (26.4)
South	1,431 (28.6)
West	1,526 (30.4)
Unknown	13 (0.3)
Charlson comorbidity index; mean (SD), median	0.5 (1.1), 0.0
Chronic disease score; mean (SD), median	2.1 (2.5), 1.0

*CDHP* consumer-driven health insurance, *COMP* comprehensive medical insurance, *EPO* exclusive provider organization, *HMO* health maintenance organization, *POS* point of service, *PPO* preferred provider organization.

Of the initial 5,012 patients who started a monotherapy SSRI, 30.7% (*n* = 1,545) of patients continued their therapy with no change in the 1-year post-index period (Table 2). These continuation patients had an average of 8.6 medication refills and a mean (SD) length of therapy of 9.3 (2.3) months. Add-on pharmacotherapy was prescribed to 37.8% of the cohort. These add-on patients continued their index SSRI, with an average of 7.8 refills and a mean (SD) length of therapy of 7.7 (3.1) months for the index drug. Another 15.2% of patients were switched from their initial SSRI therapy to a different non-SSRI medication. These switch patients only refilled their index SSRI an average of 2.9 times, with a mean (SD) length of therapy of 3.1 (2.7) months before changing therapy. Discontinuation of the initial SSRI without further observed pharmacological intervention occurred for 16.0% of the cohort. For these patients, the mean (SD) length of therapy was 3.6 (2.4) months, and they had 3.3 refills on average.

**Table 2:** Frequency distribution of second-step intervention by initial monotherapy SSRI treatment

**Second-step intervention**	**Initial monotherapy treatment**						
	**Citalopram hydrochloride**	**Sertraline hydrochloride**	**Escitalopram oxalate**	**Fluoxetine hydrochloride**	**Paroxetine hydrochloride/mesylate**	**Fluvoxamine**	**Total**
	***N*** **= 1,510**	***N*** **= 1,380**	***N*** **= 936**	***N*** **= 899**	***N*** **= 272**	***N*** **= 15**	***N*** **= 5,012**
	***n *** **(%)**	***n *** **(%)**	***n *** **(%)**	***n *** **(%)**	***n *** **(%)**	***n *** **(%)**	***n *** **(%)**
Continued	469 (31.1)	425 (30.8)	295 (31.5)	286 (31.8)	66 (24.3)	4 (26.7)	1,545 (30.8)
Discontinued	266 (17.6)	207 (15.0)	137 (14.6)	145 (16.1)	43 (15.9)	4 (26.7)	802 (16.0)
Switch	254 (16.8)	206 (14.9)	138 (14.7)	120 (13.3)	48 (17.6)	5 (33.3)	771 (15.4)
Add-on	521 (34.5)	542 (39.3)	366 (39.1)	348 (38.7)	115 (42.3)	2 (13.3)	1,894 (37.8)

Among the patients who added a second class of medication to their SSRI therapy (*n* = 1,894, 38%), the most commonly prescribed add-on medication classes were anxiolytics (40.2%) and antidepressants (37.1%), with the most common being bupropion (15.4%) or tricyclics (predominantly trazodone hydrochloride) (15.8%). For patients who switched from their SSRI therapy (*n* = 771, 15%), the most commonly prescribed second-line classes were serotonin norepinephrine reuptake inhibitors (SNRIs) (25.3%), bupropion (24.1%), and benzodiazepines (17.9%) (Table 3).

**Table 3:** Frequency of different medication classes within second-step switch or add-on treatments

**Medication class**	**Second-step switch**	**Second-step add-on**
	**(*N*** **= 771)**	**(*N*** **= 1,894)**
	***n *** **(%)**	***n *** **(%)**
Antidepressants		
SNRI	195 (25.3)	76 (4.0)
Bupropion	186 (24.1)	292 (15.4)
Tricyclic antidepressants	53 (6.9)	299 (15.8)
Other antidepressants^a^	27 (3.5)	35 (1.8)
** Total (antidepressants)**	**461 (59.8)**	**702 (37.1)**
Anxiolytics		
Benzodiazepine	138 (17.9)	727 (38.4)
Buspirone	10 (1.3)	34 (1.8)
** Total (anxiolytics)**	**148 (19.2)**	**761 (40.2)**
Anticonvulsants	47 (6.1)	106 (5.6)
Lithium	4 (0.5)	6 (0.3)
Second-generation antipsychotics		
Quetiapine	15 (1.9)	43 (2.3)
Aripiprazole	9 (1.2)	65 (3.4)
Other second-generation antipsychotics^b^	2 (0.3)	45 (2.4)
** Total (antipsychotics)**	**26 (3.4)**	**153 (8.1)**
Stimulants	10 (1.3)	35 (1.8)
Norepinephrine reuptake inhibitor	3 (0.4)	10 (0.5)
Two-drug combination		
Anxiolytic + antidepressant	33 (4.2)	42 (2.2)
Second-generation AP + antidepressant	8 (1.0)	15 (0.8)
Two antidepressants	9 (1.2)	9 (0.5)
Second-generation AP + anxiolytic	0 (0)	15 (0.8)
Other	16 (2.1)	35 (1.9)
** Total two-drug combinations**	**66 (8.6)**	**116 (6.12)**
Combination of three or more drugs	6 (0.8)	5 (0.3)

*AP* antipsychotic. ^a^Other antidepressants include nefazadone hydrochloride and mirtazapine; ^b^other second-generation antipsychotics include olanzapine, risperidone, and ziprasidone.

Although there were some differences among the absolute frequencies of second-step interventions as provided by different specialty physicians (Table 4), the ordering of the frequency of second-step interventions followed a similar pattern across medical specialties. Within each physician specialty, except for doctors of medicine (MDs) (not elsewhere classified) and physicians in multispecialty groups, add-on was the most frequent second step, followed by continuation, discontinuation, and switching. For unspecified MDs and physicians in multispecialty groups, continuation was the preferred first option. Rates of add-on therapy were highest in patients being seen by physicians who were specialists in internal medicine and obstetrics/gynecology (both 44%), and rates of switch were highest in patients whose physicians were in family practice (19%) or in multispecialty physician practices (17%).

**Table 4:** Distribution of second-step treatments by physician specialty

**Second-step intervention**	**Psychiatry**	**Family practice**	**Internal medicine**	**Multispecialty group**	**OB/GYN**	**MD**	**Unknown**	**Other**
	***N*** **= 1,403**	***N*** **= 1,090**	***N*** **= 504**	***N*** **= 339**	***N*** **= 165**	***N*** **= 336**	***N*** **= 769**	***N*** **= 406**
	***n *** **(%)**	***n *** **(%)**	***n *** **(%)**	***n *** **(%)**	***n *** **(%)**	***n *** **(%)**	***n *** **(%)**	***n *** **(%)**
Continued	487 (34.7)	306 (28.1)	137 (27.2)	117 (34.5)	37 (22.4)	119 (35.4)	215 (28.0)	127 (31.3)
Discontinued	210 (15.0)	186 (17.1)	73 (14.5)	44 (13.0)	29 (17.6)	60 (17.9)	139 (18.1)	61 (15.0)
Switch	176 (12.5)	209 (19.2)	72 (14.3)	59 (17.4)	27 (16.4)	44 (13.1)	116 (15.1)	68 (16.7)
Add-on	530 (37.7)	389 (35.7)	222 (44.0)	119 (35.1)	72 (43.6)	113 (33.6)	299 (38.9)	150 (36.9)

Overall, the cohort experienced very few MDD-related inpatient hospitalizations or emergency room (ER) visits. Across the entire cohort, 331 patients (6.6%) were hospitalized for MDD-related diagnoses, while 63 (1.3%) had MDD-related visits to an ER. Among the patients who switched therapy, the annualized mean number of outpatient MDD-related visits was 13.7 visits (SD = 13.2). Patients who added pharmacotherapy experienced more MDD-related outpatient visits, with a mean number of 15.2 (SD = 14.9). Mean annualized visits for patients who continued index therapy (mean = 11.6, SD = 12.01) or who discontinued index therapy (mean = 12.5, SD = 11.3) were slightly lower.

Table 5 presents the annualized health-care costs among patients, by second-step treatment intervention. In general, patients who added to or switched from their initial SSRI therapy had higher annualized costs across both MDD-related and non-MDD-related services compared with patients who continued with or discontinued from their initial SSRI monotherapy. There were few statistical differences in annualized costs between patients who continued versus those who discontinued therapy during the year.

**Table 5:** Mean (SD) annualized health-care costs (in dollars) for patients by second-step interventions

	**MDD-related**			**Non-MDD-related**			**Overall**		
	**Inpatient**	**Outpatient**	**ER**	**Total MDD**	**Inpatient**	**Outpatient**	**ER**	**Total non-MDD**	
	**Mean (SD)**			**Mean (SD)**			**Mean (SD)**		
Continued	165 (1,980)	1,163 (1,496)	5 (77)	1,333 (2,567)	1,129 (6,949)	3,790 (8,701)	158 (674)	5,077 (12,608)	7,916 (14,008)
Discontinued	490 (5,252)	1,207 (1,532)	16 (154)	1,714 (5,519)	1,441 (8,015)	4,735 (16,492)	250 (752)	6,426 (20,217)	9,263 (23,459)
Switch	635 (4,036)	1,594 (2,909)	17 (202)	2,246 (5,644)	2,401 (12,878)	5,103 (8,306)	313 (1,011)	7,817 (18,516)	12,189 (20,863)
Add-on	1,074 (6,873)	1,859 (4,171)	21 (324)	2,954 (8,390)	2,701 (19,719)	6,235 (13,461)	365 (1,494)	9,301 (26,426)	14,514 (30,044)
Wilcoxon *P* value	A, B, C, D, E	A, B, C, D, E	C, E, F	A, B, C, D, E, F	B, C, D, E	B, C, D, E	C, D, E, F	B, C, D, E	A, B, C, D, E

*A* difference between switched and added cohort is statistically significant (*P* < 0.05), *B* difference between switched and discontinued cohort is statistically significant (*P* < 0.05), *C* difference between switched and continued cohort is statistically significant (*P* < 0.05), *D* difference between added and discontinued cohort is statistically significant (*P* < 0.05), *E* difference between added and continued cohort is statistically significant (*P* < 0.05), *F* difference between discontinued and continued cohort is statistically significant (*P* < 0.05).

## Discussion

Consistent with findings from clinical effectiveness trials, this study suggests that approximately a third of patients who are treated with an SSRI will continue with monotherapy, while the majority of patients will require a second-step intervention. Adjunctive or add-on therapy was favored as a second-step intervention as compared with switching, and this pattern was observed across different medical specialties. The most common second-step interventions observed in this claims-based analysis were benzodiazepines and tricyclic antidepressants (most commonly trazodone). One hypothesis is that the add-on therapy is being used for management of residual symptoms. Insomnia, fatigue, anxiety, and cognitive symptoms are frequent residual symptoms associated with MDD [14], and they can also be adverse events associated with SSRI therapy [15]. Benzodiazepines are likely used for their anxiolytic effects, and trazodone may be prescribed for sleep-related symptoms. Similarly, a common use for bupropion is to augment antidepressant effects, but it may also be used to manage treatment-emergent sexual dysfunction [16]. Interestingly, despite regulatory approval as add-on therapy for patients with inadequate response to an antidepressant during the time period studied, there was limited use of the second-generation antipsychotics.

While this study was restricted to patients who initiated therapy on SSRI medications only, Schulz and Joish [17] conducted a US claims study examining second-step interventions among 7,273 patients with MDD who initiated *any* antidepressant therapy from 2002 to 2006. In their sample, 40.3% experienced a switch following their initial antidepressant, 1.5% had an add-on medication, and 58.2% maintained their initial antidepressant monotherapy. Another similar study was conducted by Milea et al. [18]. In this US claims analysis of 134,287 adults and children prescribed new antidepressant treatment in 2004–2006, 23.2% experienced a change in their treatment. The most frequent changes were switch to another antidepressant (9.5%) and combination with another antidepressant (9.1%). Augmentation with an antipsychotic, anticonvulsant, or lithium added an additional 4.2% of patients to the combination therapy group, totaling 13.3% receiving add-on therapy. In Europe, a retrospective cohort study of medical records from 2008 to 2009 was conducted in Spain [19]. One advantage of this study was the availability of patient information regarding response to the therapy. In this work of 2,260 patients, 43% of patients were classified as having an inadequate response to their initial monotherapy antidepressant treatment and were treated with a switch (43.2%), an additional AD (15.5%), a dose increase (14.6%), or continuation (26.7%) [19].

The differences in the frequencies of second-step treatment interventions between the current study and the above studies may be related to the study period. Each study examined claims from years prior to the 2007 FDA approval of the antipsychotic aripiprazole for the indication of the treatment of patients with inadequate response to antidepressant monotherapy or prior to the EU approval of adjunctive treatment for MDD. The advent of the approval of aripiprazole along with a subsequent approval of quetiapine as an add-on therapy for MDD has garnered greater attention to both the unmet medical needs of patients with MDD and the therapeutic option of add-on treatments [20]. Another difference among studies is that these prior studies only included medications classified as antidepressants as potential switch or add agents, whereas the present study captured a broader range of psychotropic drug classes commonly used in the treatment of patients with MDD and thus increased the coverage for add-on therapy.

Notably, approximately a third of patients in this analysis continued with their treatment with monotherapy, and only 15% discontinued pharmacological treatment altogether. The rate of continuation is consistent with expectations that approximately one third of patients may be expected to remit with monotherapy [5]. The rates of continuation may be also related to the inclusion requirement that patients have at least two codes of MDD. In a previous database study based on health records from general practitioners, one of the stronger predictors of duration of treatment following new antidepressant treatment was whether or not a depressive illness was coded in the record [21].

With regard to costs, as might be expected, patients who required second-step interventions of either add-on therapy or switching were associated with higher total health-related costs, possibly due to a more difficult to treat illness. Previous work has demonstrated that patients who require a switch generally present with a more acute depressive illness that may include more previous episodes of depression, comorbid psychiatric disorders, and/or concurrent prescriptions of anxiolytic or hypnotic medications [22]. Both US and international studies have demonstrated that patients who have a partial or non-response, and who require additional treatment, are more costly compared with patients who achieve remission with an initial treatment intervention [23,24].

The findings presented here should be interpreted in the context of the limitations of the study design. First, this study was conducted using an administrative claims database and included only patients with medical and prescription benefit coverage. As a result, the results may not generalize well to other clinical populations. Second, as the medical claims database does not include patient assessments, the use of diagnostic codes served as a proxy and may not be as rigorous as direct diagnostic assessments for identifying individuals with MDD. Third, without measurement of patient response, the reasons behind the selection of second-step interventions cannot be ascertained. Fourth, this study did not examine the medication dosage, which may also be a factor in treatment selection. For example, quetiapine in low doses is used primarily to ameliorate insomnia symptoms, whereas higher doses are indicated for antidepressant efficacy. Recent publications have highlighted the great variability in dosing of second-generation antipsychotics in the depressed population and thus blurs these objectives [25,26]. Finally, while the indirect costs of MDD have been found to be substantial [3], this analysis focused exclusively on direct medical costs.

The strengths of this study include the overall size of the sample and the real-world generalizability of data drawn from a broad claims database. The findings reinforce the medical needs of patients with MDD as prescription patterns suggest that the majority of patients who are prescribed an SSRI will need additional pharmacotherapy. Further, the findings highlight the necessity for physicians across different specialties to be aware that second-step treatments are to be expected in the treatment of MDD. With this perspective, physicians and patients can partner together more confidently to individualize treatment to achieve the goal of remission.

## Competing interests

SB and ED are employees and shareholders of Eli Lilly and Company. PC is a former employee and a current shareholder of Eli Lilly and Company.

## Authors’ contributions

PC conceived the study and conducted an earlier version of these analyses. ED participated in the revision of the design and execution of this study. ED and SB interpreted all the results. All authors participated in preparation of the draft and read and approved the final manuscript.
